# The River Past, God Forgotten, and the Ferryman Punished

**Published:** 1919-05-17

**Authors:** 


### THE RIVER PAST, GOD FORGOTTEN, AND THE FERRYMAN PUNISHED.

IN the old and well-nigh forgotten days of the horse omnibus, a wayfarer received a stinging cut from the whip of the driver of one of these antique vehicles. The injured person swung round and hit the conductor a mighty blow with an umbrella. Justice was perhaps a little too blind on the occasion, and most of us would consider that it fell short of perfection, but the method is not yet completely obsolete. Something of the same kind has taken place at the British Farmers' Hospital at Brussels, concerning which a paragraph recently appeared in THE HOSPITAL (April 26, p. 93).

The ways of the pedagogue are crude and simple. At a certain school the effigy of the pious founder, or of a former headmaster—it was a long time ago, and it does not matter which—was found one morning adorned with a cocked hat made of newspaper, and with a common clay-pipe skilfully attached to the mouth. Justice demanded condign punishment for the offender, but upon whom was it to be visited? The headmaster assembled the boys, and with awful solemnity called upon the offender to declare himself. Either the offender was not present, or he was so certain of being led to immediate execution that he preserved an obstinate and nefarious silence, and the outrage remained for years a mystery. But the methods of pedagogues do not change, and the method of discovering a culprit, a method scarcely worthy of Mr. Sherlock Holmes, is still sometimes employed. It has in fact been employed quite recently by the O.C. the British Farmers' Hospital. He called all the sisters before him and demanded the name of her who had sent to THE HOSPITAL the article on "The River Past and God forgotten." It happened that none of them had sent the article, so none could own up; but one of them declined to shelter herself under a technicality, and owned that she had groused in a private letter to a friend about their salaries being three months in arrear. But sisters should not grouse, even in private letters to friends, about a trifle like that; so the sister in question was dismissed the service. Thus the conductor was punished for the fault of the driver, for the driver, who wrote that article, and is writing this, is not at all concerned at the action of the O.C. Even the conductor is not very severely punished, for she escapes from a service in which her salary is three months overdue to a service in which it will be punctually paid. It is the other sisters who are being punished, and if the O.C. had not had his judgment obscured by his indignation, he would have seen that it would have been a much more severe punishment to keep the erring sister to the end of her engagement without pay than to send her home to Blighty, to which she and most of her colleagues are longing to escape. In any case, he will never succeed in intimidating THE HOSPITAL by dismissing his nurses.

However, martyrdom seldom fails to advance the cause of the martyr, and so it is in this case. Before the article that was the cause of the sister's dismissal appeared in THE HOSPITAL, not only was no pay given, but no advance could be obtained. It was "quite impossible." Now an advance has been offered. It is time that these salaries were paid, and they must be paid. The central authority of the British Farmers' Hospital does not appear to know of the delay, for on one of the sisters applying to headquarters, she received immediately a confession of ignorance that the payments had not been made, an expression of regret, and a cheque for the full amount due. The fault evidently lies with some intermediary, but still the central authority is responsible, and the intermediary should be brought to book at once. It would only be fitting if the sisters who have been kept so long without their money were compensated by being dismissed the service and sent back to Blighty, while the culprit or culprits whose fault it is that their subordinates have remained so long unpaid should be retained in their posts with salaries permanently three months in arrear. What is the O.C. doing, and what is the matron doing that headquarters are kept in ignorance and the sisters are kept without pay? Whether more sisters are dismissed or not, these questions will be pressed until they are answered.

